# Scan Time Reduction of PLCs by Dedicated Parallel-Execution Multiple PID Controllers Using an FPGA

**DOI:** 10.3390/s22124584

**Published:** 2022-06-17

**Authors:** Gnanasekaran Dhanabalan, Sankar Tamil Selvi, Miroslav Mahdal

**Affiliations:** 1 Department of Electronics and Communication Engineering, AAA College of Engineering and Technology, Sivakasi 626123, India; dhanabalan@aaacet.ac.in (G.D.); stsece@nec.edu.in (S.T.S.); 2 Department of Control Systems and Instrumentation, Faculty of Mechanical Engineering, VSB-Technical University of Ostrava, 17. Listopadu 2172/15, 708 00 Ostrava, Czech Republic

**Keywords:** analog to digital conversion, data acquisition, field programmable gate arrays, PI control, programmable logic controller, scan time

## Abstract

A programmable logic controller (PLC) executes a ladder diagram (LD) using input and output modules. An LD also has PID controller function blocks. It contains as many PID function blocks as the number of process parameters to be controlled. Adding more process parameters slows down PLC scan time. Process parameters are measured as analog signals. The analog input module in the PLC converts these analog signals into digital signals and forwards them to the PID controller as inputs. In this research work, a field-programmable gate array (FPGA)-based multiple PID controller is proposed to retain PLC scan time at a lower value. Concurrent execution of multiple PID controllers was assured by assigning separate FPGA hardware resources for every PID controller. Digital input to the PID controller is routed by the novel idea of analog to digital conversion (ADC), performed using a digital to analog converter (DAC), comparator, and FPGA. ADC combined with dedicated PID controller logic in an FPGA for every closed-loop control system confirms concurrent execution of multiple PID controllers. The time required to execute two closed-loop controls was identified as 18.96000004 ms. This design can be used either with or without a PLC.

## 1. Introduction

The contribution of a programmable logic controller (PLC) in an automation system is significant. It acts as a controller for closed-loop system(s) and executes combinational or sequential logic repeatedly through the ladder diagram (LD). It is capable of handling a sufficient number of analog and digital signals. However, adding more and more signals to it is a burden. The PLC needs an analog input module (AIM), an analog output module (AOM), and a proportional, integral, and derivative (PID) function block in the LD to realize a closed-loop control system. This ensures that the output of the closed-loop control system is always at the desired value. Whenever the PLC reads the PID function block in its LD, it refers to the AIM to read the analog value concerned with that function block. It generates a digital output after completing the execution of the PID function block and forwards it to the AOM. A digital to analog converter (DAC) in the AOM converts this digital output into an analog signal and activates the final control element. Adding more PID function blocks delays the execution of the remaining rungs in the LD and increases the scan time of the PLC. The basic reason behind this is that the PLC, AIM, and AOM have processors to perform their functions.

An elaborate literature review was carried out to understand how researchers focus on the multiple closed-loop control systems. A model for composition and inventory control for a continuous ethanol–water nonlinear pilot distillation column has been developed in [1]. A computer acts as a controller, and the control action is established using a neural network combined with the genetic algorithm. A novel technique to monitor the condition of a wind turbine gearbox using an NI data acquisition card and LabVIEW is explained in [2]. The design introduced a new signal processing technique to monitor the condition of the gearbox. A digital signal processor (DSP)-based speed control action for a three-phase induction motor using the vector-controlled method has been implemented in [3]. The DSP processor modifies the value of the phase current to vary the motor speed. A water pumping system has been developed in [4]. It used a PLC and an industrial wide area network (WAN) to control the water level in the tank. The digital implementation of a fast, discrete Stockwell transform (FDST) for accurate power quality (PQ) event detection and energy metering is discussed in [5]. The FDST algorithm was implemented in an FPGA, and the PQ events were analyzed using LabVIEW. The design used a 12-bit analog to digital converter (ADC), available in the FPGA board, to convert the test analog signal into digital. A PLC-SCADA-based control system for a water storage tank is discussed in [6]. It used an RSLOGIX 5000 PLC in its design. The speed control of a three-phase induction motor using a PLC has been implemented by [7]. The PLC maintains the speed of the induction motor at the desired value. An idea of detecting induction motor faults using FPGAs is discussed in [8]. It used a 16-bit serial output ADC to convert the acquired current signal into a digital signal.

The design of a parallelized multi-PID controller using a field programmable gate array (FPGA)-based multiprocessor is discussed in [9]. It used an enhanced PicoBlaze microcontroller (EPM). The design was tested with four PID controllers that ran simultaneously. However, this work did not discuss how this multiple PID controller handles analog signals. An example cited in the work does not require the conversion of analog signals into digital. Therefore, it is not clear whether this design can perform the concurrent execution of multiple PID controllers when an analog signal is to be maintained at the desired set point. Thus, the review indicates that single or multiple closed-loop control systems have been developed using a PLC, computer, DSP, or FPGA, or a combination of these pieces of equipment. 

The FPGA has being identified as a better alternative that can perform the function of a processor [10] in an improved way. One such example is the design of the FPGA-based floating-point processor discussed in [11]. FPGAs can also be used in the implementation of PID controllers [12,13]. The design of an ultrasonic local positioning system using an FPGA for robot applications is discussed in [14]. It used a PID controller so that the robot could track the line accurately. Researchers are eager to find an optimized solution for PLCs in terms of FPGAs [15]. Researchers have also started to realize PID controllers using FPGAs, as discussed in [16]. This work proposed a method in which more than one closed-loop control system will be concurrently processed by the dedicated hardware resources of the FPGA without compromising the scan time of the PLC.

## 2. Overview of Closed-Loop Control System with PLC

In the early 1990s, researchers developed a dedicated hardware controller that did not demand any software. The ASIC design of a PI controller is discussed in [17]. Realization of a multiple PID controller using a low power single chip has been discussed in [18]. Conventionally, a closed-loop control system has a controller, final control element, and transmitter. The controller compares the desired value with the transmitter output and actuates the final control element to maintain the process variable at the desired value. 

**Figure 1:**
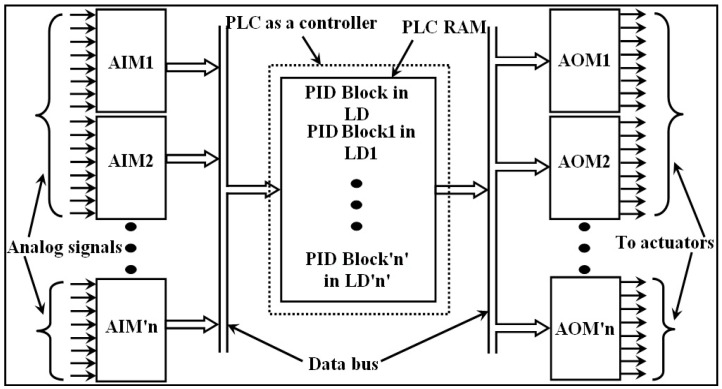
Organization of closed-loop control system using a PLC.

The controller employs a microprocessor/microcontroller [19] to realize controller algorithms such as P/PI/PID. It has an inbuilt ADC to convert the analog output of the transmitter signal into digital. A single closed-loop control system needs an ADC, a microprocessor as a controller, and a DAC. This count increases along with the number of closed-loop control systems. However, a single microprocessor shall be defined as a controller. In a practical scenario, all the ADCs and DACs are developed as AIMs and AOMs, respectively. The processor in the PLC is used to execute the control algorithm, as shown in Figure 1.

Both the AIM and the AOM must have a processor to ensure proper signal conversion. Thus, the AIM, AOM, and PLC have processors in their architecture [20]. Because the processor executes only one instruction at a time, the PLC has to wait for the AIM to complete its operation. The PID function block in the LD is ordered as last in the execution of a ladder diagram. Including PID function blocks in the LD creates an unnecessary delay in the execution of digital signals. If there is a device that takes care of PID function blocks, then the burden of the PLC can be greatly reduced.

This work has proposed a solution of multiple PID controllers developed using an FPGA that can be configured as a mixture of AIM, DIM, AOM, and DOM. The present configuration of a PLC-based automation system does not have the configuration of AIM/AOM/DIM/DOM in a single module.

## 3. Design of Single and Multiple PID Controllers

Section 3.1. and Section 3.2. elaborate the design of single and multiple PID controllers developed using an FPGA. Section 3.1.1 explains the design of an ADC that ensures concurrent conversion of more than one analog signal into digital. As a whole, this chapter provides a novel solution for the simultaneous processing of all PID controllers.

### 3.1. FPGA Design of PID Controller

A PID controller helps to maintain the value of a process parameter at the desired value. It requires three parameters, proportional gain, integral time, and derivative time, to be adjusted to achieve better performance. In this work, VerilogHDL code has been developed for the PID controller. If required, the PID controller can be easily changed into a PI controller by assigning the derivative time as zero in HDL (Hardware Description Language) code [21]. The standard equation of a PID controller is given as:

(1) $$u\left(t\right)=k_{P}[e\left(t\right)+\frac{1}{T_{I}}\overset{t}{\underset{0}{\int }}e\left(t\right)dt+T_{D}\frac{de\left(t\right)}{dt}]$$

In Equation (1), e(t) is the error signal, u(t) is the controller output, $k_{P}$ is the proportional gain, $T_{I}$ is the integral time, and $T_{D}$ is the derivative time. Because the equation must be realized by an FPGA, it is converted into the form of a difference equation [22,23], as mentioned in Equation (2).

(2) $$u\left[k\right]=u\left[k-1\right]+k_{1}e\left[k\right]+k_{2}e\left[k-1\right]+k_{3}e\left[k-2\right]$$

where,

$$k_{1}=k_{p}(1+k_{i}+k_{d})$$

$$k_{2}=-k_{p}(1+2k_{d})$$

$$k_{3}=k_{p}k_{d}$$

Inputs and outputs to and from the multiple closed-loop control system are multiple. The execution becomes concurrent when separate hardware is assigned to execute Equation (2) for every input. Hence, multiple closed-loop control systems designed using FPGAs can be visualized as real multiple input and multiple output systems. 

#### 3.1.1. Single Closed-Loop Control System—Proposed

**Figure 2:**
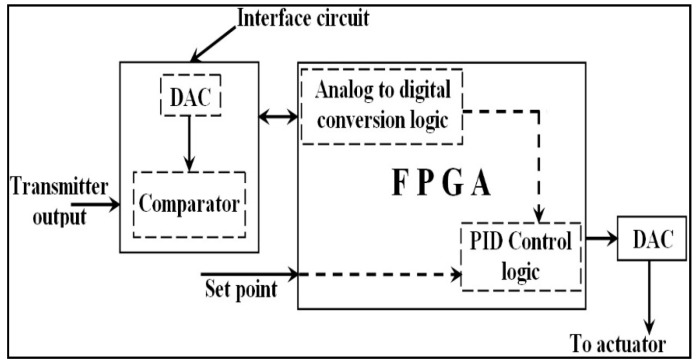
FPGA design of single closed-loop control system.

In a closed-loop control system, inputs to a PID controller are set to point to the transmitter’s output, wherein the calculation of the control error (difference of these inputs) is realized in-side the controller. The transmitter receives input from a sensor. An industrial standard transmitter generates output in the range of 0–5 V. The output of the transmitter is an analog signal. The interface circuit that comprises a DAC, comparator, and analog to digital conversion logic in the FPGA, as in Figure 2, converts the transmitter’s analog signal into digital. The working principle of successive approximation register (SAR) type ADC is preferred in many applications, as mentioned in [24].

**Figure 3:**
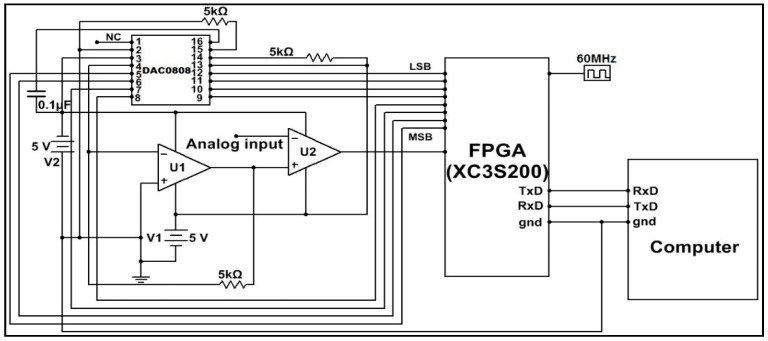
FPGA design of analog input module for a single channel.

This work has proposed a novel conversion process of analog signals into digital, which can be seen in Figure 3. It was developed, simulated, and verified using Multisim [25]. 

It functions on the working principle of successive approximation register (SAR) type ADC. The most significant bit (MSB) of the SAR register will be set, and the remaining bits will be in reset condition. A register in an FPGA is declared as a SAR register. The DAC converts this SAR content into analog, and a comparator compares it with the analog signal that is to be converted into digital. Because the DAC output is connected to the non-inverting input of the comparator, the MSB will be retained as such, provided the DAC output is greater than the analog input signal. Otherwise, it will be reset. 

Setting or resetting the SAR content will be completed by the FPGA. The FPGA then sets the consecutive MSB bit leading towards the least significant bit (LSB) and repeats the above procedure. Because all the bits have to be verified in this manner, an 8-bit ADC will require eight clock pulses.

The equivalent digital output of the analog signal can be identified by reading the content of the SAR register at the eighth clock pulse. It can also be read by a computer through RS232 serial data communication. This helps to establish a data acquisition system (DAS).

The conversion time, *τc*, of the proposed design is computed based on the settling time of DAC0808 (*τs*), the delay time of the comparator (*τcd*), and the delay time of the FPGA logic (*τfd*). The digital output of the ADC in this work is 8-bit. Hence, *τc* is calculated from the following Equation (3):

(3) τc=8×(τs+τcd+τfd)

In general, for an n bit ADC, Equation (2) is modified to:

(4) τc=n×(τs+τcd+τfd)

where n indicates the number of clock cycles required to complete the conversion.

Thus, the generated digital output is stored in the FPGA register. The PID control logic shown in Figure 2 accepts this register content as its process variable. Therefore, *e*(*t*) is generated by subtracting the setpoint from the value of the process variable. PID control logic generates *u*(*t*) and forwards it to the final control element (FCE). Usually, the FCE accepts analog signals. Hence, the digital output of the PID controller is converted into analog using DAC. 

Real-time implementation of a closed-loop control system has used DAC0808, IC741, and XC3S200 FPGA chips. The comparator was designed using IC741. The clock signal applied to the Xilinx XC3S200 FPGA chip was 50 MHz. 

#### 3.1.2. Multiple Closed-Loop Control System—Proposed

**Figure 4:**
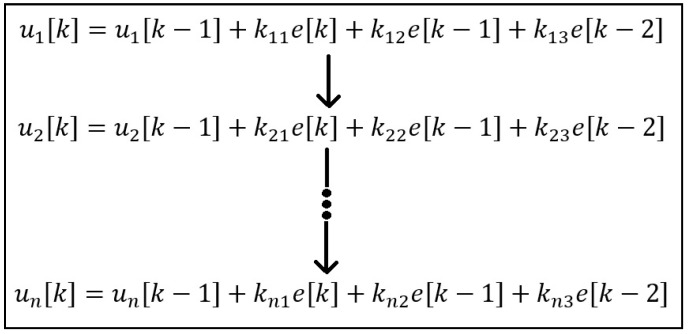
Multiple PID controller equation for microprocessor.

Conventionally, the microprocessor processes multiple closed-loop control systems. However, it sequentially executes all the closed-loop systems, as mentioned in Figure 4.

**Figure 5:**
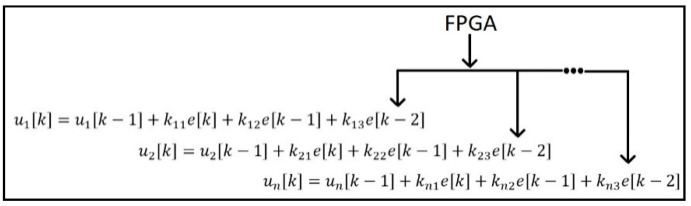
Multiple PID controller equation for FPGA.

One of the major drawbacks of this system is that the processor will be able to execute its nth PID controller equation at nth µs, assuming the time required to execute one PID controller is 1 µs. The proposed system was used to developed a multiple PID controller by which all PID controller equations will be executed simultaneously, as in Figure 5. The parallel processing capacity of an FPGA discussed in [26] indicates the possibility of realizing multiple PID controllers using FPGAs. 

**Figure 6:**
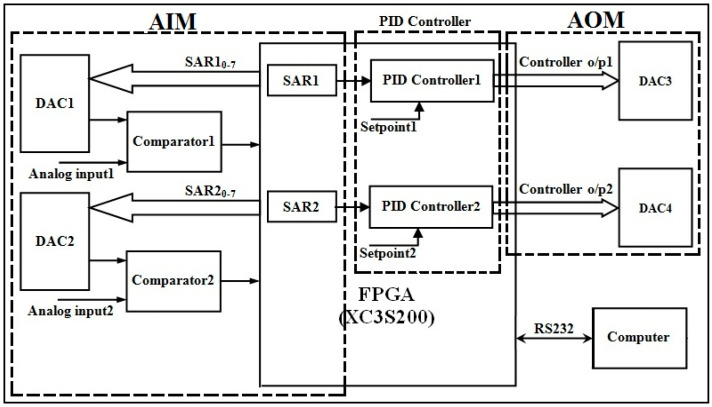
Closed-loop control system for two analog signals.

This is achieved by assigning separate PID controller logic and hence separate FPGA hardware resources for each closed-loop control system. Assigning hardware for each PID controller does not demand any novelty. A perfect multiple closed-loop control system can be established when all the PID controllers can simultaneously access its desired value in the form of a digital signal. This work has resolved the issue by integrating the ADC conversion process with the PID controller, as in Figure 6. By comparing Figure 6 with Figure 2, one can understand that a multiple PID closed-loop control system is established with a comparator, two DACs, and PID controller logic for every closed-loop control system.

Figure 6 shows the design that can concurrently maintain two process parameters at their desired values. The concept of AIMs evolved when a set of DAC0808 and a comparator is included for every closed-loop control system. Similarly, the concept of AOMs evolved when a DAC0808 is included in every closed-loop control system. In the same way, individual PID control logic is adopted in FPGAs for every closed-loop control system. Thus, the FPGA plays the role of the PLC. It should be noted that Figure 6 is evolved from Figure 2 and Figure 3. Thus, the novelty of this work is identified in Figure 6. It has an AIM, an AOM, and a PID controller. The maximum number of closed-loop control systems that can be realized in an FPGA is based on its hardware resources.

This work has developed VerilogHDL code for eight closed-loop control systems, which have used approximately 55% of the FPGA hardware resources.

### 3.2. Case Study of Multiple PID Controller—Level and Pressure Process Station

The design of multiple closed-loop control systems was tested and verified for two closed-loop control systems. The design depicted in Figure 5 was used to maintain the desired value of level and pressure of the level and pressure process stations, respectively.

#### 3.2.1. Level Process Station

**Figure 7:**
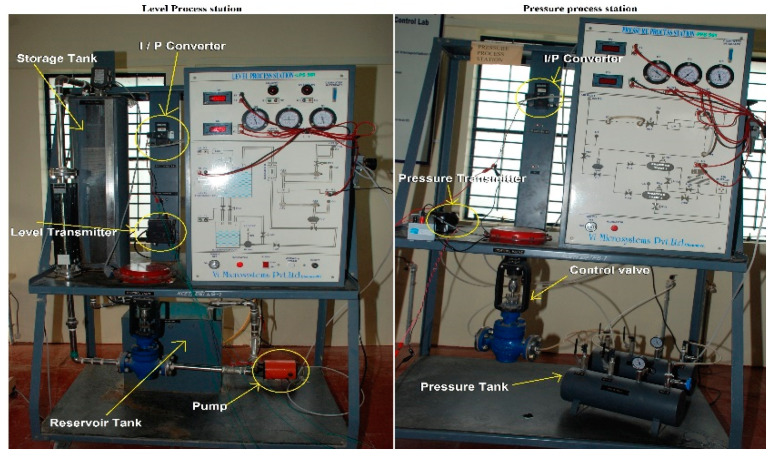
Level process station.

The Laboratory set-up of the level and pressure process station used to test the closed-loop control system is shown in Figure 7. A pump located below the storage tank pumps water from the reservoir tank. It has two outlets. One is connected to a storage tank and the other to the reservoir. 

**Figure 8:**
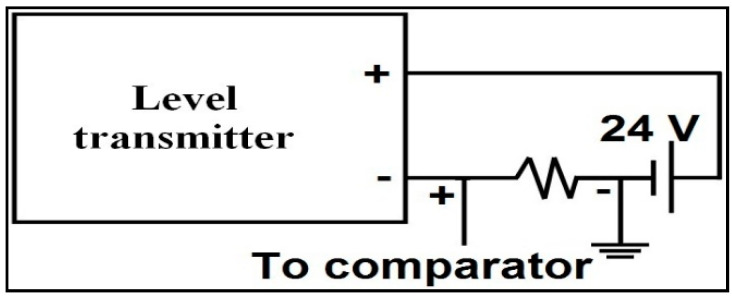
Conversion of the output current of the transmitter into voltage.

The control valve is installed in between the pipeline of the pump and the storage tank. A capacitance probe sensor is used to sense the water level. Its output is converted into 4 to 20 mA current signal by a level transmitter. The connection between the transmitter and the non-inverting terminal of the comparator is shown in Figure 8. An industrial standard transmitter generates an output of 4 to 20 mA current signal (Sunita Sinha [27]). A resistor is connected in series with the 24 V DC power supply to convert the 4 to 20 mA current signal into a 1 V to 5 V voltage signal. An equivalent resistor that adopts the conversion of current signal into voltage was identified as 240 Ω. The output of the FPGA is connected to the I/P converter.

A resistor connected in series with the DAC and I/P converter ensures that the current flowing in the loop is in the range of 4 to 20 mA. DAC output is set as 5 V. The resistor value is adjusted and found to be 130 Ω when the current flow in the circuit is 20 mA [28].

#### 3.2.2. Pressure Process Station

The laboratory setup of the pressure process station in Figure 7 has two storage tanks to store compressed air. It is possible to store compressed air, either in both the tanks or only in one tank, by operating the hand valve interlinked between the two tanks. The maximum pressure that can be stored in the tank is 2 $\mathrm{Kg}/{\mathrm{cm}}^{2}$. As discussed in the level process station, resistor values connected to the pressure transmitter and I/P converter were identified as 240 Ω and 130 Ω, respectively. 

#### 3.2.3. FPGA Interfacing Board

**Figure 9:**
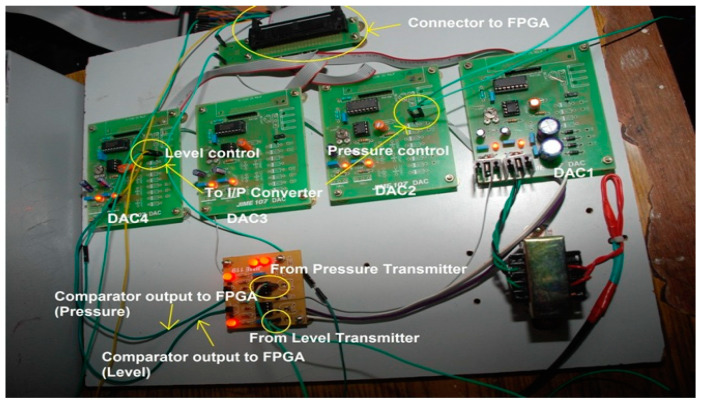
Interface board for ADC conversion, DAC output to I/P converter.

An FPGA interface board is designed to process two process parameters: level and pressure. Each parameter requires two DACs and one comparator. One DAC and a comparator are used to convert the analog signal of the transmitter output into digital. A second DAC is used to activate the I/P converter for which the output is initiated from the FPGA as a controller output. Hence, the interface board has four DAC0808 chips and two comparators, which were designed using IC741, as shown in Figure 9. 

DAC1 and DAC2 are used for the pressure process station, and DAC3 and DAC4 are used for the level process station. The outputs of comparators and inputs to the DACs are integrated into a single 50-pin connector so that the other end of the connector can be connected to the FPGA board (XC3S200). This board has one 40 pin connector, three 25 pin connectors, and one universal asynchronous receiver transmitter (UART) connector. Additionally, it has eight switches and eight light emitting diodes (LEDs). 

### 3.3. VerilogHDL Code—Acquisition, Control, UART

The contribution of FPGAs in the design of closed-loop control systems is crucial. They are used to convert an analog signal of transmitter output into digital, initiate control actions based on the transmitter output, and transfer necessary data to the computer for monitoring.

#### 3.3.1. VerilogHDL Code—Single PID Controller, UART

FPGA implementation of any design can be carried out by representing a problem in HDL code. This is verified for its proper functionality during simulation. Thus, verified code is converted into a bit file after synthesis for FPGA implementation. This work has used VerilogHDL code to develop the entire logic for the FPGA implementation of multiple closed-loop control systems. 

The essential elements of VerilogHDL code for a PID controller is listed below. The main module controller invokes the modules convert and transmit. The convert module is responsible for converting the analog signal into a digital signal based on the logic discussed in Section 3.1.1. This digital output is applied as an input to the PID controller. Hence, the logic to generate controller output has also been included in this module. Thus, generated controller output (outputtocontroller) and digital output (datatodac), combined into data, are transmitted to the computer by executing the module transmit. This performs the function of UART data transmission. Clock pulse time, clk, of the FPGA is reduced to ‘clkdivided’, which is the required clock pulse for ADC conversion. Similarly, FPGA speed is reduced in the module to cope with the speed of the computer. Because separate hardware resources are assigned for the modules convert and transmit, UART data transmission does not affect the function of ADC conversion and PID controller.

**Figure 10:**
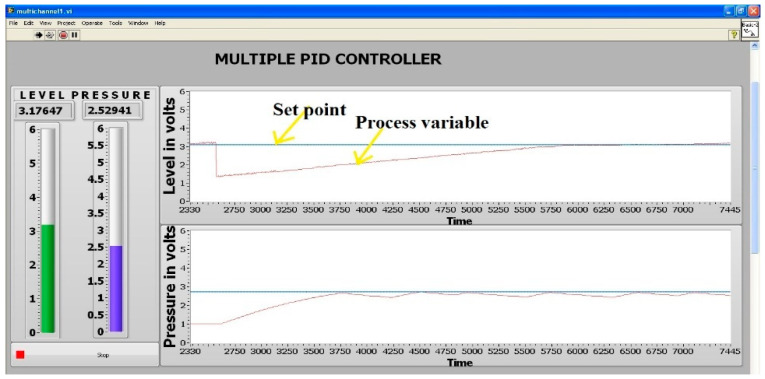
Data acquisition using LabVIEW.

In the transmit module, the respective baud rate and the parity bit are set as 19,200 and even. The transmission packet is designed as 11-bit data that has a start bit (1), parity bit (1), and stop bit (1). LabVIEW, a graphical programming language, is normally used for data acquisition in industrial automation [29]. LabVIEW code was written to acquire the data from the FPGA. Figure 10 displays the setpoint and process parameter values of level and pressure. It can be seen that level is being maintained at the setpoint, and the pressure value oscillates between the set point and its lower value, because both the controllers were tested with the same $K_{p}$, $K_{i}$, and $K_{d}$ values.

#### 3.3.2. VerilogHDL Code—Multiple PID Controller

An inherent feature of FPGAs helps to achieve the design of multiple PID controllers. The main module instantiates the module adcconversion for every addition of a controller so that a multiple closed-loop control system is established. For example, if the number of closed-loop control systems to be designed is eight, then the module adcconversion will be instantiated eight times. 

This ensures that all eight instantiations will be executed concurrently and independently. For the case study, the adcconversion module is instantiated twice. Instantiating a module in VerilogHDL needs the declaration of inputs and outputs only. This creates the feature that the addition or deletion of a PID controller can be carried out in a single instruction.

## 4. Results and Discussion

This work was aimed at designing a multiple PID controller using devices that are readily available in the market and can be integrated into the design requirements. As mentioned in Section 3, DAC0808, IC 741, and XC3S200 (SPARTAN3E family of FPGA) were used to test two closed-loop control systems. This section discusses the delay time created by the complete design by calculating the delay time created by DAC0808, IC 741, and the FPGA logic. Finally, the delay time is compared with the existing PLC, AIM, and AOM to ensure that the proposed design performs better and helps to reduce the scan time of the PLC.

### 4.1. Performance Analysis of Closed-Loop Control System

Even though multiple closed-loop control systems for two process parameters were implemented using the SPARTAN3 family of Xilinx FPGA, FPGA logic for up to eight closed-loop control systems was synthesized using the SPARTAN3E (XC3S1600E-4-FG484), SPARTAN6 (XC6SLX100T-4-CSG484), and VIRTEX5 (XC5VLX330T-2-FFT1738) family of Xilinx FPGA devices.

**Figure 11:**
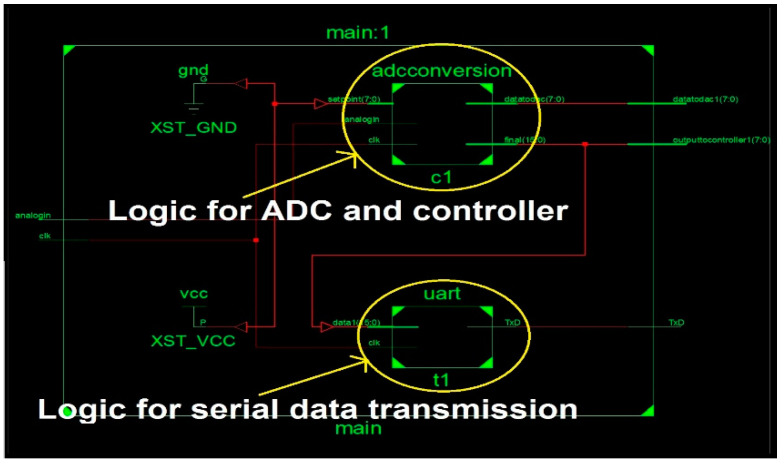
RTL Schematic for single closed-loop control system.

Figure 11 shows the register transfer logic (RTL) schematic of FPGA logic synthesized for the single closed-loop control system. It indicates that separate hardware resources are allocated for the closed-loop control system and serial data transmission logic. The assignment of separate hardware for ADC conversion and UART transmission in Figure 11 ensures that conversion can be performed when UART hardware is transmitting data to the computer.

#### 4.1.1. Hardware Resources of FPGA

Table 1, Table 2 and Table 3 show the synthesized results of utilization of hardware resources of an FPGA when the number of closed-loop control systems is varied from 1 to 8. This complete logic was synthesized for XC3S1600E-4-FG484, XC6SLX100T-4-CSG484, and XC5VLX330T-2-FFT1738 FPGA devices. It should be noted that the logic is meant for ADC conversion, UART transmission, and the PID controller. The highest percentage of FPGA resource utilization to realize the logic has been identified as 54.48%. However, it is not the same for all the hardware resources of the FPGA. Many of the resources are utilized in less than 25%. Table 3 proves that only a lesser amount of hardware resources is used as it is the higher version of FPGA.

Thus, it is evident that unutilized hardware resources can be used to perform other suitable operations. The hardware resources and I/O pins (960 pins) of XC5VLX330T-2-FFT1738 indicate that it is possible to establish a 16 closed-loop control system in a single FPGA.

#### 4.1.2. Delay Analysis of FPGA

It is necessary to identify the time required by the FPGA to execute the logic for the ADC conversion, PID controller, and UART data transmission. Delay time created by different FPGA chips for different numbers of closed-loop control systems is shown in Table 4. 

It shows that delay time is maintained at constant, even when the number of the closed-loop control system is more than one. The lowest and the highest delay times are identified as 4.026 ns (XC5VLX330T-2-FFT1738) and 18.58 ns (XC3S1600E-4-FG484), respectively. It is also evident that delay time reduces in proportion to a higher version of FPGA.

#### 4.1.3. Delay Analysis of DAC and the Comparator

**Figure 12:**
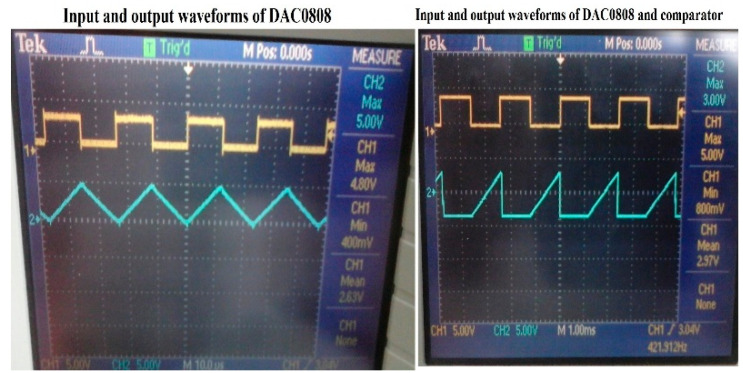
Input and output waveforms of DAC0808, Comparator.

The Delay time of DAC0808 was identified by applying a minimum and maximum of 0 V and 5 V, respectively. The settling time of DAC0808 under this condition was analyzed by applying a square wave signal with varying frequencies. As shown in Figure 12, the settling time was found to be 0.0235 ms. The FPGA activates DAC0808 eight times to complete the conversion, and hence the total delay time by DAC0808 is 0.1881 ms. 

Because DAC0808 output is connected to the comparator circuit, the delay time created by the comparator when it is connected to DAC0808 was analyzed by applying a square wave signal with varying frequencies, and the delay time for eight cycles was identified to be 18.96 ms. A snapshot of comparator output (CH2) for the applied square wave at the input of DAC0808 (CH1) is shown on the right-hand side of Figure 12. The total delay time of 18.96 ms remains constant, even when the number of closed-loop control systems is eight, because each closed-loop control system will be equipped with a comparator and DAC0808.

#### 4.1.4. Delay Analysis of Single and Multiple Closed-Loop Control System

The delay time of a single closed-loop control system includes a delay time of the FPGA and comparator circuit with DAC0808. The delay time of the XC3S1600E-4-FG484 SPARTAN3E FPGA device is 17.278 ns. It comes to 138.224 ns because the FPGA logic is executed eight times to complete the conversion. The delay time of the comparator circuit with DAC0808 has already been found to be 18.96 ms. Hence, the delay time of a single closed-loop control system is 18.96000015 ms. Therefore, the operating frequency of this proposed design is 53.76 kHz. The cumulative delay time calculation for the different number of closed-loop control systems with different types of FPGA is listed in Table 5. 

The delay time of the comparator with DAC0808 suppresses the delay time of FPGA logic because it is in ms while the latter is in ns. Additionally, it is shown to be 18.96 ms because there will be a separate comparator and DAC0808 for each closed-loop control system. Hence, the approximate delay time of any number of closed-loop control systems is 18.96 ms. However, actual delay time slightly varies in ns, as represented in Table 5, with the type of FPGA and is maintained at constant when the number of closed-loop control systems is more than one.

### 4.2. Performance Comparison of the Proposed Design with PLC

Microcontroller and PLC-based closed-loop control systems have been referred to as a benchmark to validate the proposed FPGA-based multiple closed-loop control system. 

Table 1 in [19] shows the control module’s execution times of temperature control, lighting control, and oxygen control, which are 31.195 ms, 31.035 ms, and 31.006 ms, respectively. Hence, the execution time of a single controller is identified as 124.171 ms. The execution time of two closed-loop control systems in the proposed design (18.96000015 ms) is less than the above microcontroller-based closed-loop control system. A data logger for a physiological signal recording in [30] employed a PLD as a controller and an ADC to convert the physiological signal into digital. Its operating frequency is represented as 11.5 kHz. Compared to this, the operating frequency of the proposed design (53.76 kHz) is higher.

The performance of the proposed work was also compared with the latest Allen Bradley PLC. The module input scan time of the eight-channel analog input module 1756-IF8 is 16 ms [31]. The execution time of the PID functional block diagram (FBD) by ControlLogix5580 is 6.887µs [32]. When the number of PID FBD is eight, the execution time becomes 55.096 µs (6.887 × 8). The module scan time of the eight-channel analog output module 1756-OF8 is 8 ms [31]. Therefore, the scan time of the PLC, ControlLogix5580, to execute eight closed-loop control systems is the summation of the module I/O scan time and the execution time of PID FBD, which is 24.055 ms. This indicates that the proposed design performs better and greatly reduces the scan time of the PLC when 1756-IF8, 1756-OF8, and PID FBD are replaced by the proposed design.

In the case of the PLC, it is to be noted that the PID function is part of the LD program. As mentioned in [33], the sweep time of a Series 90–30, model 331 PLC will be 12.611 ms when it has 700 Boolean instructions, 300 output coils, and 200 math functions in an LD with five 16-point input modules and four 16-point output modules. This model allows PID function execution once in 10 ms. Assuming that a PID function is included in the aforementioned example, based on the nature of the application, it shall be included anywhere in the LD, and it should be in the LD in such a way that it is called for execution at 7 ms. However, it will not be executed because the minimum time required for execution is 10 ms. Hence, the function will be executed during the next iteration, which is at 14 ms. Hence, the PID function will be executed once every 14 ms. If the PID function is included after the completion of the aforementioned example, then it will be executed once every 12.611 ms. It is evident that PID execution by a PLC is not only based on its own execution time, but also on the number of rungs in the LD.

As stated earlier, the execution time for FPGA-based single and multiple closed-loop control systems is 18.96000014 ms. Thus, the design gives better performance when compared with the existing system.

## 5. Conclusions

This work shows that the proposed design is able to reduce the scan time of PLCs when realizing PID controllers using this approach. As per industrial standards, the outputs of the transmitter and controller are 4–20 mA and 1–5 V, respectively. Therefore, this work has considered this fact and developed the design accordingly. The impact of using DAC0808 and IC741 in the proposed design proves that the design aids in reducing the scan time of PID controllers. However, it should be noted that the design will provide higher execution speed when these components are replaced by high-speed devices. This proposed design can be used independently or in combination with PLCs. The total number of PID controllers required in an application decides the number of FPGAs used in the design of multiple PID controllers. When the design is to be used with a PLC, the concept of networking must be included in the FPGA. This is also applicable when there is a situation in which the content of one FPGA is to be shared with other FPGAs. Hence, the authors have aimed to develop multiple PID controllers that support network communications in the future.

## Figures and Tables

**Table 1:** Utilization of FPGA hardware resources for different numbers of closed-loop control systems using XC3S1600E-4-FG484 (SPARTAN3E).

Hardware Resource Name	Number of Closed-Loop Control Systems								Available Hardware Resources
	Hardware Resources Used								
	1	2	3	4	5	6	7	8	
Number of Slices	112	242	359	468	583	693	807	916	14,752
Number of Slice flip flops	107	213	310	406	502	599	695	792	29,504
Number of 4input LUTs	200	429	635	832	1029	1221	1422	1613	29,504
Number of bonded IOBs	19	36	53	70	87	104	121	138	376
Number of MULT18X18SIOs	2	4	6	8	10	12	14	16	36
Number of GCLKs	2	3	4	5	6	7	8	9	24
Resource Utilization in percentage (%)	0.76	1.64	2.43	3.17	3.95	4.7	5.47	6.21	
	0.36	0.72	1.05	1.38	1.7	2.03	2.36	2.68	
	0.68	1.45	2.15	2.82	3.49	4.14	4.82	5.47	
	5.05	9.57	14.1	18.62	23.14	27.66	32.18	36.7	
	5.56	11.11	16.67	22.22	27.78	33.33	38.89	44.44	
	8.33	12.5	16.67	20.83	25	29.17	33.33	37.5	

**Table 2:** Utilization of FPGA hardware resources for different numbers of closed-loop control systems using XC6SLX100T-4-CSG484 (SPARTAN6).

Hardware Resource Name	Number of Closed-Loop Control Systems								Available Hardware Resources
	Hardware Resources Used								
	1	2	3	4	5	6	7	8	
Number of Slice Registers	93	167	241	314	387	461	534	608	126,576
Number of Slice LUTs	131	271	389	505	633	738	863	977	63,288
Number of fully used LUT-FF pairs	87	155	222	290	357	427	492	559	1026
Number of bonded IOBs	19	36	53	70	87	104	121	138	296
Number of BUFG/BUFGCTRLs	2	3	4	5	6	7	8	8	16
Number of DSP48A1s	2	4	6	8	10	12	14	16	180
Resource Utilization in percentage (%)	0.07	0.13	0.19	0.25	0.31	0.36	0.42	0.48	
	0.21	0.43	0.61	0.8	1	1.17	1.36	1.54	
	8.48	15.11	21.64	28.27	34.8	41.62	47.95	54.48	
	6.42	12.16	17.91	23.65	29.39	35.14	40.88	46.62	
	12.5	18.75	25	31.25	37.5	43.75	50	50	
	1.11	2.22	3.33	4.44	5.56	6.67	7.78	8.89	

**Table 3:** Utilization of FPGA hardware resources for different numbers of closed-loop control systems using XC5VLX330T-2-FF1738 (VIRTEX5).

Hardware Resource Name	Number of Closed-Loop Control Systems								Available Hardware Resources
	Hardware Resources Used								
	1	2	3	4	5	6	7	8	
Number of Slice Registers	107	197	286	374	462	551	639	728	207,360
Number of Slice LUTs	138	243	354	460	570	675	784	892	207,360
Number of fully used LUT-FF pairs	87	152	219	285	354	418	484	551	1069
Number of bonded IOBs	19	36	53	70	87	104	121	138	960
Number of BUFG/BUFGCTRLs	2	3	4	5	6	7	8	9	32
Resource Utilization in percentage (%)	0.05	0.1	0.14	0.18	0.22	0.27	0.31	0.35	
	0.07	0.12	0.17	0.22	0.27	0.33	0.38	0.43	
	8.14	14.22	20.49	26.66	33.12	39.1	45.28	51.54	
	1.98	3.75	5.52	7.29	9.06	10.83	12.6	14.38	
	6.25	9.38	12.5	15.63	18.75	21.88	25	28.13	

**Table 4:** Delay time analysis of FPGA logic for different numbers of closed-loop control systems with different FPGAS.

	Delay Time in ns							
Number of Closed-Loop Control Systems	1	2	3	4	5	6	7	8
XC3S1600E-4-FG484 (SPARTAN3E)	17.278	18.58	18.58	18.58	18.58	18.58	18.58	18.58
XC6SLX100T-4-CSG484 (SPARTAN6)	12.655	12.576	12.576	12.576	12.576	12.576	12.576	12.576
XC5VLX330T-2-FF1738 (VIRTEX5)	4.026	5.418	5.418	5.418	5.418	5.418	5.418	5.418

**Table 5:** Total delay time of multiple closed-loop control system.

Number of Closed-Loop Control Systems	1	2	3	4	5	6	7	8
Delay time of comparator with DAC0808 (ms)	18.96	18.96	18.96	18.96	18.96	18.96	18.96	18.96
FPGA logic delay time (XC3S1600E-4-FG484 (SPARTAN3E)) (ns)	138.224	148.64	148.64	148.64	148.64	148.64	148.64	148.64
Total delay time of closed-loop control system (ms)	18.96	18.96	18.96	18.96	18.96	18.96	18.96	18.96
FPGA logic delay time (XC6SLX100T-4-CSG484 (SPARTAN6)) (ns)	101.24	100.608	100.608	100.608	100.608	100.608	100.608	100.608
Total delay time of closed-loop control system (ms)	18.96	18.96	18.96	18.96	18.96	18.96	18.96	18.96
FPGA logic delay time (XC5VLX330T-2-FF1738 (VIRTEX5)) (ns)	32.208	43.344	43.344	43.344	43.344	43.344	43.344	43.344
Total delay time of closed-loop control system (ms)	18.96	18.96	18.96	18.96	18.96	18.96	18.96	18.96

## Data Availability

Not applicable.
